# Psychism

**Published:** 1889-12

**Authors:** 


### PSYCHISM.

"The Society for Psychical Research will probably take note of the case of the poor Steeple Jack, William Bishop, in Birmingham, whose wife beheld his tragic death in a dream on the night before the fatal occurrence. According to the widow's statement at the inquest, which there is no reason to doubt, it was on Tuesday night last, she dreamt that she had just taken to her husband his breakfast, and was engaged in watching him at his perilous work of repairing a chimney shaft at a great height, when she saw a hook come out of the brickwork of the chimney and the deceased fell. On waking, she told her husband the little story; and when he was going out to his labor the next morning, her last words were: 'Be careful, Bill; you know what I dreamt.' As a fact, the accident did not occur by the coming out of a hook, but by the accidental tipping up of a plank, by which Bishop was unhappily precipitated from a great height. The coincidence, however, is sufficiently remarkable to impress those who believe that dreams are not the mere *disjecta membra* of the day's thoughts and impressions. Yet, what more simple than Mrs. Bishop's dream? Antonio's friend confessed that had he a precious argosy at sea, he could not see 'the sandy hour-glass run,' but he would 'think of shallows and of flats.' Well might William Bishop's loving wife in like manner brood upon the dangers of the steeple and the sky-pointing factory chimney shaft till her thick-coming fancies followed her into the mystic realm of sleep. The famous marvel of Maria Martin, and the 'murder in the old Red Barn,' which after sixty years is still referred to by Suffolk folk with breathless awe, is found on a careful examination of the evidence at the trial of Corder to be susceptible of a like simple explanation."—*London Telegraph.*

